# Enhanced Li^+^ Elution and Adsorption by Tannin-Modified Hydrophilic-H_2_TiO_3_ Titanium–Lithium Ion Sieve

**DOI:** 10.3390/ma19153181

**Published:** 2026-07-25

**Authors:** Xia-Zhong Zhang, Guang-Ming Cheng, Shu-Ying Liu, Ke-Jie Wang, Lei-Lei Wang, Li-Yuan Zhang, Hong-Bo Li, Ting-Xing Zhao

**Affiliations:** 1 School of Materials and Chemistry, Southwest University of Science and Technology, Mianyang 621010, China; zhangxiazhong@163.com; 2 School of Chemical and Environmental Engineering, Neijiang Normal University, Neijiang 641112, China; 18855823238@163.com (G.-M.C.); liushuying630@163.com (S.-Y.L.); 18048118123@163.com (K.-J.W.); wangleilei22311@163.com (L.-L.W.); 3 Special Agricultural Resources in Tuojiang River Basin Sharing and Service Platform of Sichuan Province, Neijiang 641112, China; 4 State Key Laboratory of Environment-Friendly Energy Materials, Mianyang 621010, China

**Keywords:** titanium–lithium ion sieve, hydrophilicity, tannin, precipitation-peptization process, elution, adsorption

## Abstract

Tannin-modified Li_2_TiO_3_ powders (TA-Li_2_TiO_3_) were prepared by a precipitation-peptization process with titanium sulfate as the titanium source, lithium acetate as the lithium source, ammonia as the precipitator, and hydrogen peroxide as the complexing agent. The effects of the tannin addition ratio and the calcination temperature on the crystal structure and surface morphology of TA-Li_2_TiO_3_ were studied. After pickling TA-Li_2_TiO_3_ with diluted hydrochloric acid, TA-H_2_TiO_3_ was obtained. Its elution behavior and lithium adsorption performance were studied and the adsorption isotherm and kinetics were investigated. The results indicated that the crystal structure was well-defined, and a uniform mesoporous surface morphology was observed on the sample surface with a calcination temperature of 750 °C and a tannin addition ratio of 1 g:1 L. The specific surface area was increased by 15.1 m^2^/g compared with the unmodified one, and the hydrophilicity of the TA-H_2_TiO_3_ significantly improved. After elution with 0.2 mol/L hydrochloric acid for 8 h, the elution equilibrium was achieved, the lithium elution ratio reached 98.15% and the lithium adsorption capacity reached 39.13 mg/g, which were significantly higher than that of the unmodified one. The adsorption isotherm is more consistent with the Langmuir model, the adsorption kinetics follows the pseudo second order model, and the adsorption mechanism is chemical monolayer adsorption.

## 1. Introduction

As the first metal element in the periodic table, lithium is the lightest. It has excellent characteristics such as active chemical properties, large specific heat capacity, low expansion coefficient, and is widely used in batteries, glass ceramics, grease, nuclear energy, etc. [1,2,3]. At this stage, it is an indispensable key raw material for the development of new energy automobile industry, and is known as “metal monosodium glutamate”. Lithium resources mainly exist in lithium mines, salt lake brine, and seawater. Although the technology of extracting lithium from ore is mature, many problems also exist, such as the cumbersome steps, high energy consumption, and large amount of waste residue. The total reserves of lithium in seawater are abundant; however, the mass concentration of lithium is relatively low (0.17 mg/L). The lithium reserves in salt lake brine account for 80% of the total land lithium resources, and the lithium content is higher than that in seawater. At present, the lithium extracted from salt lake brine accounts for the largest share of the world’s lithium carbonate production [4,5]; however, the traditional methods are inefficient. Therefore, developing new technologies for lithium extraction from liquid lithium sources such as salt lake brine and seawater is of great significance. The main methods for extracting lithium from salt lake brine include solvent extraction [6,7,8], precipitation [9], membrane filtration [10,11], electrochemical [12,13] and adsorption method [14,15,16]. From an economic and environmental perspective, adsorption is considered as the most suitable way for lithium recovery, and lithium ion sieve is one of the adsorbents that has been extensively studied.

Lithium ion sieve adsorbents mainly include manganese-based [17,18,19,20,21], aluminum-based [22,23], and titanium-based [24,25,26,27,28] lithium ion sieves. These adsorbents enable that the insertion and desorption of lithium ions can be carried out without causing any significant damage to their structures. Manganese oxide adsorbents exhibit a high adsorption capacity and selectivity for lithium ions in seawater. However, when lithium is eluted from the lithium manganese oxide (LMO) matrix in dilute acidic solution, manganese dissolves, which results in reduced adsorption capacity and poor recyclability. Aluminum-based adsorbents have disadvantages such as low adsorption capacity. Titanium-based lithium ion sieves have attracted much attention due to their outstanding advantages such as large adsorption capacity, stable structure under acidic conditions, excellent selectivity for lithium and good cycling performance. However, the improvement of their hydrophilicity and specific surface area still remains an urgent issue to be addressed. Among various strategies, surface modification using organic substances has achieved certain progress. Xiao et al. synthesized a superhydrophilic spinel-type lithium-ion sieve H_4_Ti_5_O_12_ (HTO-IV) using CTAB and F127 as dual surfactants to regulate the surface morphology and surface wettability of the precursors [29]. Qin et al. utilized four surfactants-sodium dodecyl sulfate (SDS), cetyltrimethylammonium bromide (CTAB), hydroxypropyl cellulose (HPC), and tannic acid (TA)-to etch the surface of H_2_TiO_3_, yielding adsorbents with enhanced hydrophilicity at the nanoscale [30]. Yang et al. employed the silane coupling agent (3-aminopropyl)triethoxysilane (KH550) to modify the surface of HTO via covalent grafting, thereby alleviating agglomeration, improving dispersity, and increasing surface area, which facilitated the enhancement of lithium adsorption capacity and adsorption rate constant of HTO/KH550 [31]. Chen et al. adopted glycerol as an additive to regulate the specific surface area and internal structure of the lithium-ion sieve, thereby enhancing its Li^+^ adsorption capacity [32]. Qin et al. prepared modified porous HTO-containing N-heteroatoms via surface modification with cetyltrimethylammonium chloride (CTAC). Through the combined effect of surface modification and spatial optimization, the Li^+^ diffusion into the ion sieve was accelerated and more adsorption sites were created [33]. Sun et al. employed a dual-surfactant soft-template method using F127 and hexadecylamine to prepare H_2_TiO_3_ with hydrophilic functional groups and a porous structure [34]. Inspired by these studies, we adopted tannin as a modifier for hydrophilic modification of the Li_2_TiO_3_-type lithium-ion sieve.

**Figure 1:**
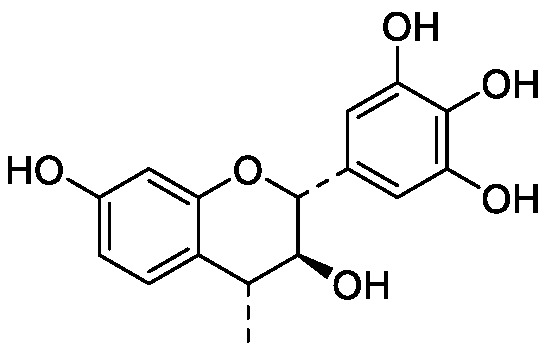
Structural formula of tannin.

Figure 1 presents the structural formula of tannin [35], which possesses abundant phenolic hydroxyl groups capable of binding metal ions to form insoluble precipitates. As a green plant-sourced biomaterial, tannic acid relies on these phenolic groups to form bonds with functional groups, serving as antioxidants, bacteriostatic agents and crosslinkers simultaneously to enhance the mechanical and physical properties of biomaterials [36]. Tannin contains abundant polyphenolic hydroxyl groups, and the polar group -OH in the polyphenolic hydroxyl structure makes it soluble in polar solutions, resulting in the hydrophily. In addition, tannin also has advantages such as low cost and easy availability. It is foreseeable that the hydrophilicity of lithium-ion sieves can be improved when tannin is used to modify lithium-ion sieves, which is beneficial for improvement of the elution rate and adsorption capacity.

In this work, the precursor of the lithium-ion sieve was prepared via an inorganic precipitation-peptization method using tannin as the hydrophilic modifier. Initially, tannin acts as a surfactant, exerting a dispersing effect that mitigates the agglomeration among adsorbent particles [30]. During the subsequent high-temperature calcination stage, tannin undergoes thermal decomposition as a soft template, resulting in the formation of a porous structure with an enlarged specific surface area, thereby exposing more adsorption sites. The enlarged specific surface area is positively correlated with increased availability of adsorption sites and enhanced affinity, which collectively contribute to the improvement of ion adsorption capacity [32].

## 2. Experimental

### 2.1. Preparation of TA-Li_2_TiO_3_

A certain amount of titanium sulfate was dissolved in distilled water, followed by the addition of concentrated ammonia water under magnetic stirring to obtain the white TiO(OH)_2_ precipitate, during which the pH of the system was adjusted to 8–9 [37]. After washing by centrifugation, it was diluted with distilled water and stirred to ensure uniformity. Then lithium acetate solution was added at a constant rate under magnetic stirring. Subsequently, a known volume of H_2_O_2_ was slowly dropped into the mixture to obtain a transparent yellow sol. After that, tannin was added to the sol at solid-to-liquid ratios of 1:1.3, 1:1, and 1:0.8 (g:L), followed by aging, drying, grinding and calcination to obtain tannin-modified Li_2_TiO_3_ precursor. According to increasing tannin content, the precursors were sequentially named TA-LTO-1, TA-LTO-2, and TA-LTO-3. The control sample without tannin doping was named LTO-0. During the calcination process, the temperature was set to 600–800 °C, and the sample was cooled in a muffle furnace.

### 2.2. Acid Washing

LTO-0 or TA-LTO and 0.2 mol/L hydrochloric acid (m/V = 1 g/100 mL) was added to an 250 mL erlenmeyer flask, and the system was treated in a water bath at 50 °C for a period of time, during which the conical flask was shaken every 2 h to avoid concentration gradients, and 4 mL of supernatant was collected at regular intervals for later use. After acid washing, the sample was filtered and washed, and then placed in an oven at 60 °C for drying to obtain lithium ion sieve(H_2_TiO_3_), named HTO-0 and TA-HTO-n (n = 1, 2, 3).

### 2.3. Adsorption

LiOH solution with a Li^+^ concentration of 2 g/L and LiCl solutions with different Li^+^ concentrations were prepared. TA-HTO-n and HTO-0 were used to adsorb Li^+^ from LiOH solution (initial Li^+^ concentration of 2 g/L) at a solid–liquid ratio of 1 g:50 mL for 24 h at 25 °C to achieve adsorption equilibrium. The supernatant was then collected, and Li^+^ content was measured using an atomic absorption spectrophotometer to calculate the adsorption capacity according to Equation (1) and to study the adsorption kinetics. The above adsorbents were employed to adsorb Li^+^ from LiCl solution with a solid–liquid ratio of 0.2 g to 60 mL to investigate the adsorption isotherm, during which the pH of the solution was adjusted with NaOH. All the adsorption experiments were performed at 25 °C.

(1) $$Q_{e}=\frac{(C_{0}-C_{e})V}{m}$$

In Equation (1) [38], *Q*_e_ is the adsorption capacity at equilibrium, mg/g; *C*_0_ represents the initial Li^+^ concentration in the solution, mg/L; *C*_e_ is the concentration of residual Li^+^ in the solution at adsorption equilibrium, mg/L; *V* is the volume, L; and *m* is the mass of the lithium ion sieve, g.

### 2.4. Characterization

The surface morphology of the samples was characterized by scanning electron microscopy (SEM, VEGA3, TESCAN, Brno, s.r.o., Brno, Czech Republic). The crystal phase composition of the samples was analyzed by X-ray diffraction (XRD, DX-2700, Dandong Haoyuan Instrument Co., Ltd., Dandong, China) using Cu Kα radiation at a scanning rate of 0.05° s^−1^ and a working voltage/current of 40 kV/40 mA. The concentration of Li^+^ was measured using an atomic absorption spectrophotometer (AAS, WFX-200, Beijing Ruili Analytical Instrument Co., Ltd., Beijing, China). A surface area analyzer (Autosorb iQ2, Quantachrome Instruments, Boynton Beach, FL, USA) was used to collect Brunauer–Emmett–Teller (BET) pore structure data and specific surface area. The valence states of the constituent elements were studied by X-ray photoelectron spectroscopy (XPS, Escalab 250 Xi, ThermoFisher Scientific, Waltham, MA, USA). The functional groups were characterized with a Fourier transform infrared spectroscopy in the range of 3500 to 400 cm^−1^ (FTIR, WQF-510A, Beijing Beifen Ruili Analytical Instrument Co., Ltd., Beijing, China). The contact angles of the samples were tested using a contact angle tester (SDCA1909002, Dongguan Shengding Precision Instrument Co., Ltd., Dongguan, China).

## 3. Results and Discussion

### 3.1. Characterization of Materials

#### 3.1.1. Effect of Tannin Addition

**Figure 2:**
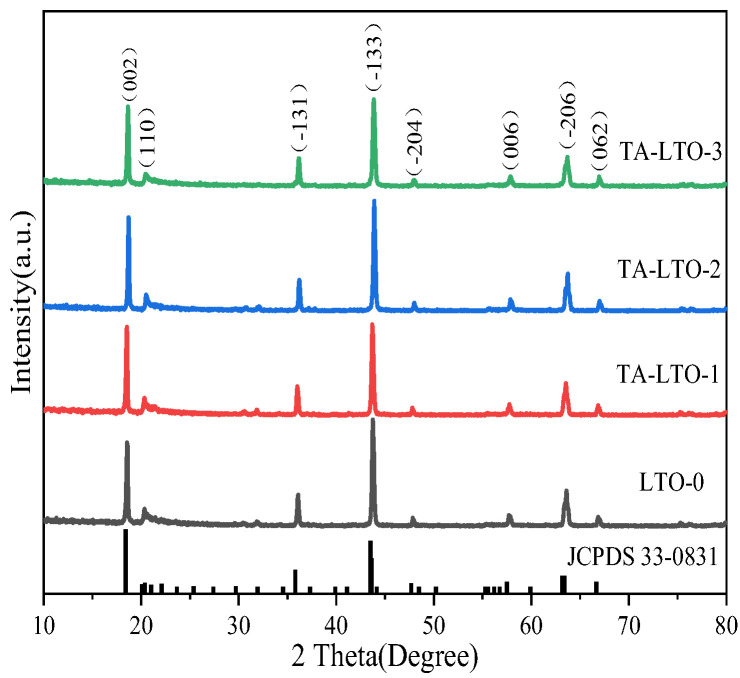
XRD patterns of Li_2_TiO_3_ modified with different solid-to-liquid ratios.

XRD patterns of TA-LTO-n were shown in Figure 2. The effect of dosage of tannin on the growth of Li_2_TiO_3_ crystals was investigated, as shown in Figure 2, in which curves a, b, c, and d, give XRD patterns of Li_2_TiO_3_ modified by adding tannin at solid–liquid ratios of 0 (no tannin, LTO-0), 1 g:1.3 L (TA-LTO-1), 1 g:1 L (TA-LTO-2), and 1 g:0.8 L (TA-LTO-3), respectively. The crystal cell parameters of the samples are basically consistent with the standard card of Li_2_TiO_3_ (ICDD PDF #33-0831). It can be observed from Figure 2 that the diffraction peak intensity of the characteristic (002) crystal plane gradually increases as the amount of tannin added increases. The diffraction intensity of crystal planes (110), (-131), (-133), (-206) initially increases and then decreases, indicating that an appropriate amount of tannin promotes precursor growth. At the same time, some low-intensity impurity peaks can be observed, possibly arising from the addition of tannin. In addition, the full width at half maximum (FWHM) of the sample peaks is narrow, and the peaks are sharp, and the intensity of the diffraction peak is strong, indicating that the samples exhibit high crystallinity and a well-developed crystal structure. And when the solid–liquid ratio is 1 g:1 L, the diffraction intensity reaches the maximum, it can be concluded that the growth of Li_2_TiO_3_ crystal is the most complete. This suggests that the modification endows the precursor with enhanced structural tolerance, which will improve its stability. In addition, it can be observed from Figure 2 that the diffraction peaks shift to lower diffraction angles(2θ) with increasing tannin addition, which is possibly due to the change in bonding mode. When the solid–liquid ratio is 1 g:1.3 L and 1 g:1 L, the shift is more pronounced, reaching a maximum at 1 g:1 L. However, the degree of shift decreases with further increase in tannin addition. The diffraction peak of the sample shifts towards a small angle, indicating that the cell parameters increase and the spacing between crystal planes increases. This expansion of the unit cell volume is consistent with Bragg’s law.

**Figure 3:**
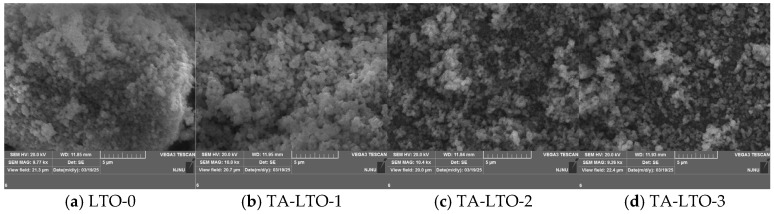
SEM images of Li_2_TiO_3_ modified with tannin for different tannin addition amounts.

Figure 3 shows SEM images of the samples before and after tannin modification with different tannin addition amounts. As shown in Figure 3a, the precursor without tannin addition exhibits a compact and dense surface with pronounced particle agglomeration, poorly defined surface features and overall poor dispersion. After the introduction of tannin (Figure 3b), the surface morphology of the precursor changes significantly, becoming porous and rough with the emergence of numerous irregular pore structures. This indicates that the addition of tannin increases the specific surface area of the precursor and improve its structural characteristics. With a further increase in tannin addition (Figure 3c,d), the surfaces gradually become rougher, the particle size decreases markedly, and the particle distribution becomes more uniform, indicating enhanced overall dispersion. However, as observed in Figure 3d, minor agglomeration occurs in specific regions, suggesting that excessive tannin addition does not further optimize the surface microstructure, and which may induce particle agglomeration and consequently compromise the microstructural integrity. Based on the morphological evolution observed from Figure 3a–d, it can be inferred that among the investigated solid-to-liquid ratios, 1 g:1 L is the optimal condition. Under this ratio, the precursor surface exhibits a moderately porous structure with homogeneously distributed pores, fine particles with a narrow size distribution and uniform morphology, and no obvious agglomeration. This is consistent with the subsequent adsorption performance experiments, which show that the best adsorption performance is achieved at a solid-to-liquid ratio of 1 g:1 L.

#### 3.1.2. Effect of Calcination Temperature

**Figure 4:**
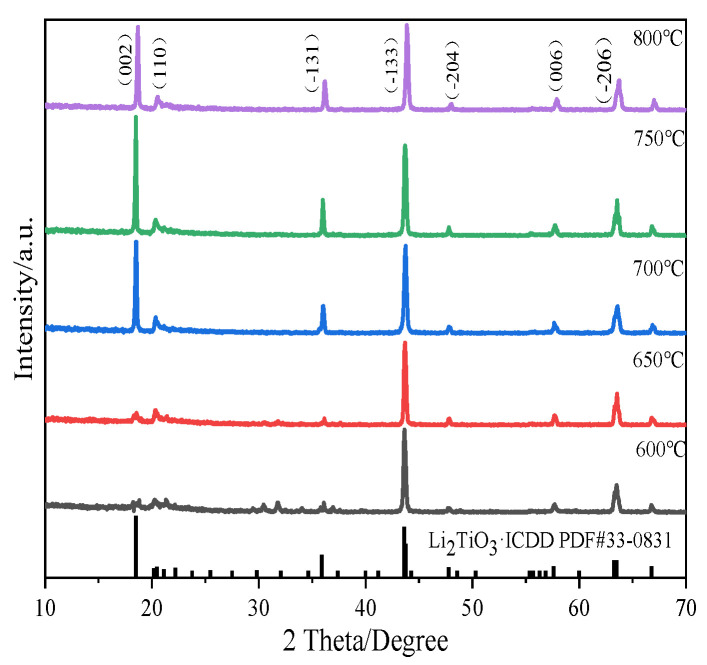
XRD patterns of TA-Li_2_TiO_3_ obtained at various calcination temperatures.

Figure 4 shows the XRD patterns of TA-Li_2_TiO_3_ calcined at different temperatures (600–800 °C) with a solid–liquid ratio of 1 g:1 L. At 600 °C, only the (-133) crystal plane exhibits a relatively strong diffraction peak, while the characteristic peaks of (002) and (-131) planes are nearly absent. The overall diffraction peaks are broad and low in intensity, indicating poor crystallinity, possible lattice defects (e.g., Li-site vacancies), and incomplete phase transformation at this temperature. With the increase in calcination temperature from 600 °C to 750 °C, all characteristic diffraction peaks gradually appear and grow in intensity. The peaks become progressively sharper, and the full width at half-maximum (FWHM) narrows continuously, demonstrating improved crystallinity, grain growth, and more complete development of the monoclinic crystal structure. Notably, the (002) and (-131) crystal planes become distinct and well-defined when the temperature reaches 700 °C. When the temperature further increases from 750 °C to 800 °C, the intensities of all diffraction peaks decrease slightly, and the sharpness of the peaks is reduced compared to the 750 °C sample. Among all tested temperatures, the sample calcined at 750 °C shows the strongest and sharpest diffraction peaks, with the best agreement with the standard PDF pattern, indicating that the crystallinity and grain growth of Li_2_TiO_3_ reach the optimal state at this temperature. Therefore, 750 °C was selected as the optimal calcination temperature for subsequent preparation processes.

**Figure 5:**
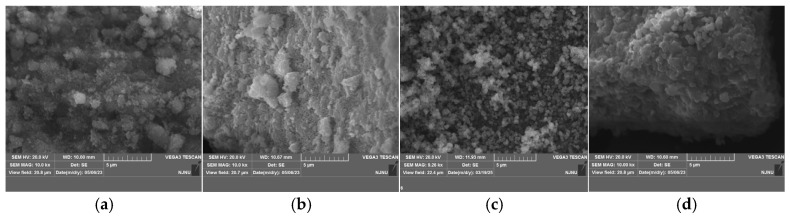
SEM images of TA-Li_2_TiO_3_ calcined at different temperatures (**a**) 650 °C; (**b**) 700 °C; (**c**) 750 °C; (**d**) 800 °C.

The morphology of TA-Li_2_TiO_3_ crystals under different calcination temperatures was characterized by SEM, shown in Figure 5. It can be observed from Figure 5a that when calcined at 650 °C, the sample surface exhibits an amorphous structure with flocculent aggregation of particles, and no well-formed crystals are developed, which is consistent with the XRD results. As the calcination temperature increases to 700 °C (Figure 5b), the flocculent morphology is drastically reduced instead of disappearing completely, the structure becomes relatively compact, and a few unevenly distributed pores appear. When the calcination temperature rises to 700 °C (Figure 5b), the flocculent morphology is drastically reduced instead of disappearing completely. The precursor tends to be densified, and a small quantity of unevenly distributed interstitial pores are formed among agglomerated particles, with a small amount of flocculent particles remaining in partial regions. With a further rise in calcination temperature, the pore density on the sample surface increases significantly. When the temperature reaches 750 °C (Figure 5c), a well-developed crystalline structure is formed, with uniformly sized and well-arranged particles on the surface, along with homogeneously distributed pores and the most abundant pore sizes. A large number of interconnected pores with consistent dimensions develop at grain boundaries, yielding the highest pore density among the four sample groups. However, when the calcination temperature is further raised to 800 °C (Figure 5d), the porous structure disappears. This is likely due to the collapse of the pore structure caused by excessively high temperature, leading to the agglomeration of particles. Such a phenomenon adversely affects the subsequent acid washing process and Li^+^ adsorption. Overall, Figure 5 indicates that at a calcination temperature of 750 °C, the sample exhibits the smallest and most uniform particle size, the highest porosity, and the most perfect crystalline structure. Under this condition, the morphology and structural characteristics are most favorable for the subsequent Li^+^ extraction and adsorption.

#### 3.1.3. Other Characterizations

**Figure 6:**
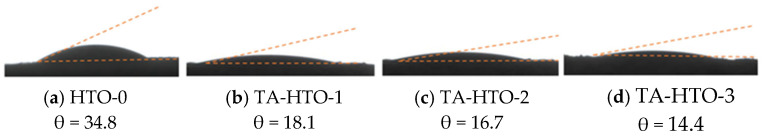
Contact angles of TA-HTO-n obtained by tannin modification with different tannin addition amounts.

To better understand the effect of tannin addition on the hydrophilicity of H_2_TiO_3_, wettability experiments were conducted. The contact angle of samples obtained with different tannin addition amounts were measured. The precursor powders were compressed into uniform sheets, and the contact angle was measured by dropping distilled water onto the sheet surface. Figure 6 shows the contact angles of the unmodified H_2_TiO_3_ and tannin-modified TA-H_2_TiO_3_ samples prepared with different tannin addition amounts. As the tannin addition increased from 1 g:1.3 L to 1 g:0.8 L, the contact angle decreased sequentially. It is well known that a smaller contact angle indicates stronger hydrophilicity. Therefore, the hydrophilicity of TA-H_2_TiO_3_ improves with increasing tannin dosage. The contact angle of the unmodified H_2_TiO_3_ is 34.8°, whereas the contact angles of TA-H_2_TiO_3_-1, -2, and -3 are 18.1°, 16.7°, and 14.1°, respectively, decreasing in this order, demonstrating that tannin effectively improves the surface hydrophilicity of H_2_TiO_3_. The improved hydrophilicity facilitates better wetting by aqueous solutions, which is beneficial for subsequent acid washing and lithium adsorption processes.

**Figure 7:**
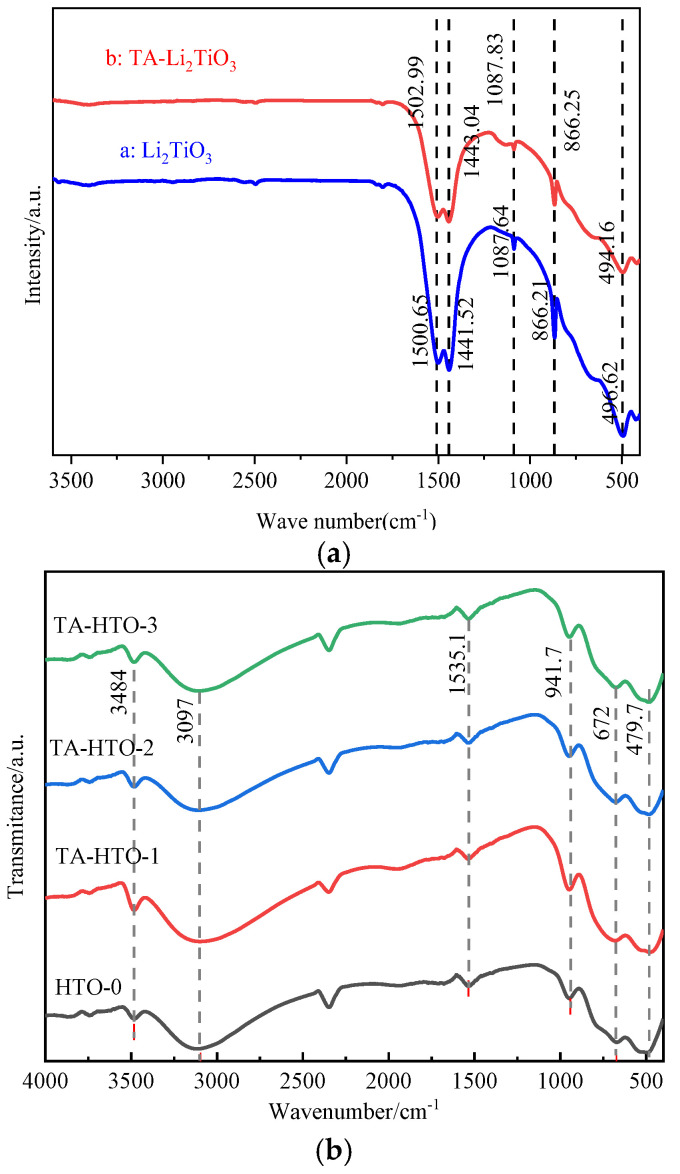
FTIR spectra of (**a**) Li_2_TiO_3_ and TA-Li_2_TiO_3_ (1 g:1 L), (**b**) HTO-0 and TA-HTO-n.

Fourier transform infrared spectroscopy (FTIR) was used to characterize the functional group structure of Li_2_TiO_3_, TA-Li_2_TiO_3_, HTO-0, and TA-HTO-n samples, and the results are shown in Figure 7. By carefully comparing the spectra of the LTO series (Figure 7a) and the HTO series (Figure 7b), it can be observed that the characteristic peaks among these samples are generally consistent, indicating that the basic functional groups have not changed significantly after tannin modification. Absorbance peaks in a wide range of 3500–3100 cm^−1^ are observed, which should be attributed to the tensile vibration of the OH group [34], and the peak of 1535 cm^−1^ may result from the bending vibration of hydroxyl-adsorbed water. In addition, the same absorption peak at approximately 866 cm^−1^ is detected in the precursor of lithium ion sieve before and after tannin modification, which attributes to the characteristic absorption peak of -O-O- (peroxy group), illustrating the formation of peroxy bonds in the sol. A vibration absorption peak near the wavelength of 1443 cm^−1^ is detected, which corresponds to a Li-O band, demonstrating the formation of Li_2_TiO_3_ crystals. Similar spectral features are observed for the HTO series, though slight variations in transmittance intensity are noted among the different treatment conditions (TA-HTO-n) compared to the untreated HTO-0.

**Figure 8:**
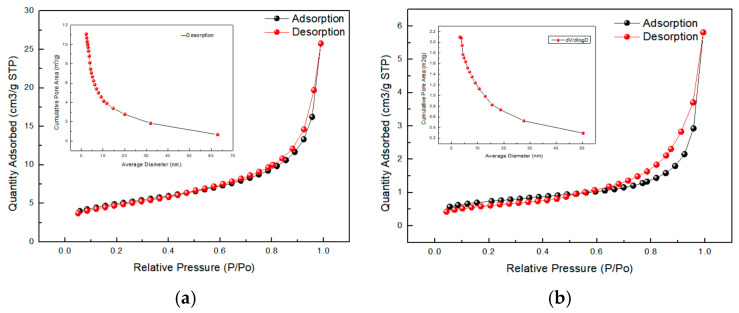
N_2_ adsorption–desorption isotherms and pore size distributions of (**a**) LTO-0 and (**b**) TA-LTO-2.

The pore structure and specific surface area of Li_2_TiO_3_ and TA-Li_2_TiO_3_ (1 g:1 L) were investigated by a N_2_ adsorption desorption isotherm (Figure 8). According to the classification of IUPAC, it can be concluded from Figure 8 that the classification of the adsorption desorption isotherm of the two samples conforms to type IV of adsorption desorption isotherm, which means that the samples before and after hydrophilic modification have mesoporous structure [34]. In addition, it can also see from the graph that the curve begins to increase slowly with a certain overlap when the relative pressure P/P_0_ > 0.05. When the relative pressure P/P_0_ > 0.1, hysteresis loops were detected, which indicates that mesoporous structure exists in the samples before and after tannin modification. From Figure 8, it can also be observed that the pore size of Li_2_TiO_3_ before and after modification is mainly distributed between 7 and 12 nm, indicating that a uniform mesoporous structure is formed.

The pore structure data of LTO-0 and TA-LTO-2 are shown in Table 1. Compared with the unmodified LTO-0, TA-LTO-2 exhibits a remarkable increase in specific surface area from 2.6 to 17.7 m^2^/g, along with an enlarged pore volume from 0.001 to 0.0073 cm^3^/g and a reduced average pore diameter from 6.46 to 5.64 nm. These changes indicate that the introduction of tannin promotes the formation of mesopores within Li_2_TiO_3_, leading to increased pore volume and decreased pore size, which collectively contribute to a substantial enhancement in specific surface area. This improvement can be attributed to the dual role of tannin: in the early stage, it mitigates particle agglomeration and refines grain size; during the subsequent high-temperature calcination, the tannin is largely decomposed, leaving behind abundant pores within the material. The larger specific surface area and well-developed mesoporous structure not only provide a greater solid–liquid contact interface and more active sites for ion exchange, facilitating the migration of Li^+^ from the solution to the material surface, but also improve the wettability and solution permeability of the material, ultimately benefiting the elution and adsorption processes.

### 3.2. Elution and Adsorption Performance

#### 3.2.1. Acid Washing and Adsorption Mechanism

β-Li_2_TiO_3_ has a layered structure, and the crystal structure formula can be written as Li[Li_1/3_Ti_2/3_]O_2_. Specifically, this structure can be expressed as cubic close-packed (ccp) oxygen ions, and metal atoms are placed in the octahedral interstices. In the Li_2_TiO_3_ crystal, Li and Ti are divided into two layers. One layer (Li layer) is only occupied by Li atoms, while the other layer (LiTi_2_ layer) is occupied by 1/3 of Li atoms and 2/3 of Ti atoms. Meanwhile, lithium atoms in the (Li) layer account for 75% of the total lithium in Li_2_TiO_3_ [39], while the remaining 25% lithium atoms are located in the (LiTi_2_) layer. The reaction activity of these two layers of lithium is different. During the acid washing process, Li located in the (Li layer) is first replaced, followed by Li located in (LiTi_2_), and in order to maintain electrical neutrality, H^+^ occupies the position of the original Li^+^. β-Li_2_TiO_3_ crystal is mainly composed of an intercrystalline layer and a main crystalline layer [31]. Chu, He and Zhang et al. [40,41,42] conducted in-depth study on the pickling mechanism, and it was found that the first pickling of Li_2_TiO_3_ would damage its crystal structure, manifested by the disappearance of diffraction peaks of the (-133) and (-206) crystal planes.

However, after the adsorption of Li^+^ by adsorbent, the disappeared diffraction peaks cannot be completely or partially restored, indicating that the binding state of adsorbed Li^+^ is different from that of Li^+^ in original Li_2_TiO_3_. The limited exchangeability of H^+^ situated in the mixed layer (LiTi_2_) leads to a practical adsorption capacity that is lower than the theoretical capacity. Even in the most favorable environment, the actual adsorption capacity of -H_2_TiO_3_ for lithium is much lower than the theoretical adsorption capacity. The pickling process can be represented by Equations (2) and (3), and the adsorption process can be expressed by Equation (4) [41].

(2) $$\;Li\left[Li_{1/3}Ti_{2/3}\right]O_{2}+H^{+}\to H\left[Li_{1/3}Ti_{2/3}\right]O_{2}+Li^{+}$$

(3) $$H\left[Li_{1/3}Ti_{2/3}\right]O_{2}+1/3H^{+}\to H\left[H_{1/3}Ti_{2/3}\right]O_{2}+1/3Li^{+}$$

(4) $$H\left[H_{1/3}Ti_{2/3}\right]O_{2}+Li^{+}\to Li^{+}\left[H_{1/3}Ti_{2/3}\right]O_{2}+H^{+}$$

Since Na^+^ is in the same main group as Li^+^, the chemical property is similar to Li^+^, and the ionic radius is larger than Li^+^. The ion-sieving effect due to size incompatibility may occur during the adsorption process, which makes Na^+^ unable to enter the lattice; however, the adsorption of Li^+^ will not be greatly affected. Thus, the ionic sieves have a special selectivity [42].

#### 3.2.2. Effect of Tannin Modification on Elution and Adsorption Performance

**Figure 9:**
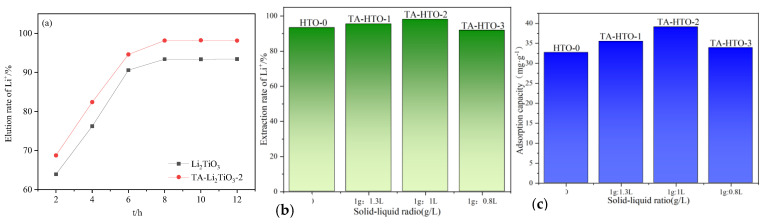
(**a**) Elution rates of Li_2_TiO_3_ and TA-Li_2_TiO_3_-2 (1 g:1 L) vs. time; (**b**,**c**) extraction rates and adsorption capacities of tannin-modified samples vs. tannin addition amount.

Figure 9a shows the elution rates of Li_2_TiO_3_ and TA-Li_2_TiO_3_-2 over time. It can be seen from Figure 9 that both Li_2_TiO_3_ and TA-Li_2_TiO_3_ can reach elution equilibrium after acid washing for 8 h, and the elution rate of TA-Li_2_TiO_3_ reached 98.15%, which was significantly higher than that of simple Li_2_TiO_3_. In addition, it can be observed that the elution rate of TA-Li_2_TiO_3_ is higher than that of Li_2_TiO_3_ at any time during the whole pickling process. This is because the surface of pristine Li_2_TiO_3_ possesses only a limited number of pores, whereas after tannin modification, the sample surface not only exhibits a uniformly distributed porous structure but also shows enhanced hydrophilicity, which facilitates Li^+^ migration. Meanwhile, the smaller particle size of TA-Li_2_TiO_3_ shortens the mass-transfer path and reduces the resistance to Li^+^ transport [33], thereby resulting in a better acid-washing effect and a higher Li^+^ elution rate. Figure 9b illustrates the extraction rate of unmodified Li_2_TiO_3_ and TA-Li_2_TiO_3_ with different tannin addition amounts at equilibrium, as well as the adsorption capacity of corresponding H_2_TiO_3_ in LiOH solution with a Li^+^ concentration of 2 g/L for 24 h. It can be observed from Figure 9b,c that the extraction rate and adsorption capacity both increase first and then decrease with the increase in tannin dosage. When the solid–liquid ratio is 1 g:1 L, the elution rate is 98.15% higher than the 93.39% of HTO-0 and the adsorption capacity reaches its maximum values, which is 39.13 mg/g. The adsorption capacity is 6.41 mg/g higher than that of the unmodified sample. The higher the elution rate, the better the adsorption capacity, and the two trends are highly consistent. This indicates that the modification does not sacrifice the ion-exchange capacity of the material; instead, it achieves more complete Li^+^ extraction and re-intercalation through structural optimization, reflecting good structural reversibility. The enhanced adsorption capacity of TA-Li_2_TiO_3_ is ascribed to the surfactant-induced increase in specific surface area, which exposes additional adsorption sites. Furthermore, the improved hydrophilicity facilitates the transport of Li^+^ ions to the adsorbent surface and to the active sites within the lithium-ion sieve, thereby boosting the Li^+^ uptake capacity of TA-Li_2_TiO_3_ [30].

The preparation of adsorbents can be seen as extracting target Li^+^ from the lattice using hydrochloric acid, accompanied by the entry of H^+^ to ensure the electrical neutrality of the solution. The exchange sites left by Li^+^ after extraction are quite narrow, but they exhibit special selectivity for accepting Li^+^ due to the lattice memory effect. Other ions can only occupy these sites if both of the following conditions are met simultaneously: similar ionic radius and approximate dehydration energy. Li^+^ is easily bound by ionic bonds, while H^+^ generally tends to form covalent interactions. Therefore, during the pickling process, as Li^+^ is removed from the lattice, H^+^ enters simultaneously to maintain electrical neutrality. However, unlike the original Li^+^, which exists in an ionic bond state, H^+^ is present in the form of electrostatic attraction.

**Figure 10:**
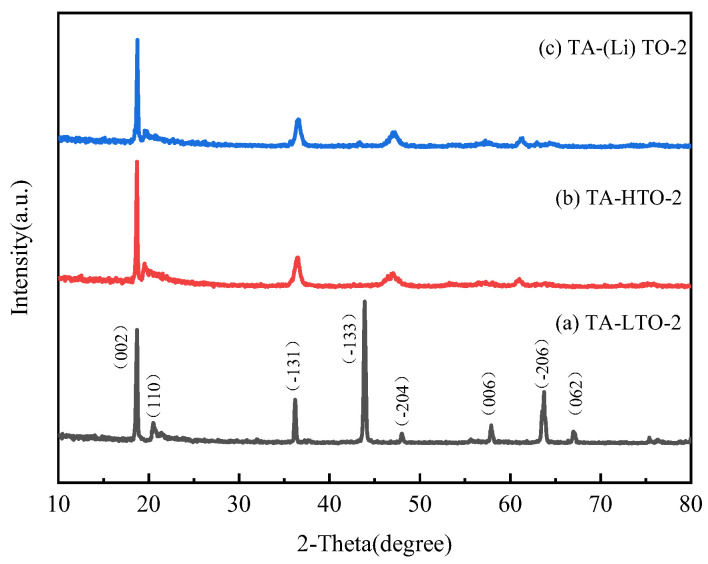
XRD patterns of crystals before pickling, after pickling and after adsorption with the solid–liquid ratio of 1 g:1 L. (**a**) TA-Li_2_TiO_3_-2; (**b**) TA-H_2_TiO_3_-2; (**c**) TA-(Li)_2_TiO_3_.

XRD was used to analyze the crystal structure changes before and after acid pickling and after lithium adsorption, and the results are shown in Figure 10. As seen, the samples before and after acid pickling and after lithium adsorption all belong to monoclinic crystals, and the basic crystal structure is maintained. Compared with the unpickled sample (Figure 10a), the characteristic diffraction peaks of (-133) and (-206) completely disappear after acid pickling (Figure 10b), indicating that most Li^+^ has been extracted and the ordered arrangement of Li^+^ in the lattice has been destroyed. The reason is that after pickling, Li^+^ is replaced by H^+^; the difference in electronegativity between H and the surrounding O is smaller than that between Li and O, so ionic bonds are no longer formed. Instead, they are combined through electrostatic adsorption, and thus the final replacement position is different from the original one.

Yu et al. [43] believe that these peaks tend to disappear due to high concentrations of stacking faults along the C-axis. By observing curve c, it can be seen that the diffraction pattern of TA-Li_x_H_2-x_TiO_3_ is almost identical to that of TA-H_2_TiO_3_, and the disappeared (-133) and (-206) peaks cannot be restored to the state of Li_2_TiO_3_ before acid washing. This indicates that during the adsorption process, lithium ions cannot be arranged in an orderly manner according to the original crystal structure of Li_2_TiO_3_. The crystal structure of the metatitanate-type lithium ion-exchanger does not change after Li^+^ adsorption, nor can it return to the crystal structure before acid washing. It also proves that the changes in crystal structure caused by acid washing are permanent and irreversible, and lithium ions do not involve any structural changes during the adsorption process [44]. Li^+^ adsorption mainly occurs on the intercrystalline layer, while H^+^ on the HTi2 layer cannot be replaced, so the corresponding diffraction peak of (-133) is still absent after adsorption, showing no significant change compared to the acid-washed sample. The adsorption behavior of the adsorbent for Li^+^ is not completely consistent with the ion exchange pathway during the acid washing process [45,46].

**Figure 11:**
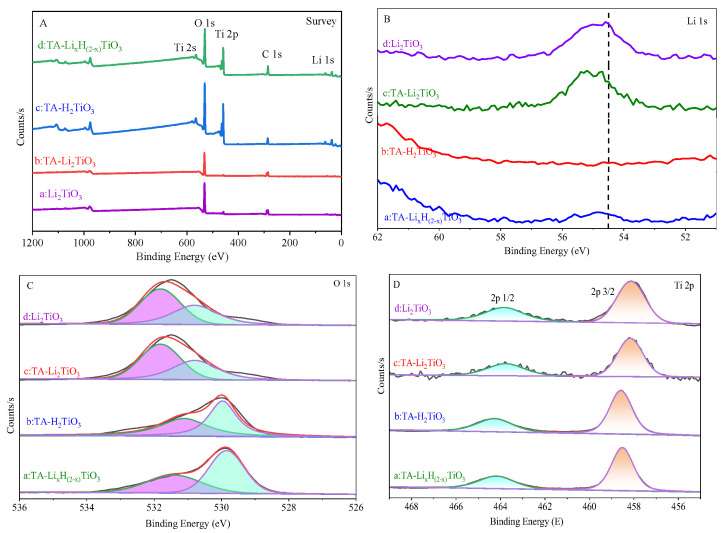
XPS images of Li_2_TiO_3_, TA-Li_2_TiO_3_, TA-H_2_TiO_3_, TA-Li_x_H_(2-x)_TiO_3_ (**A**) full spectra; (**B**) Li 1s spectra; (**C**) O 1s spectra; (**D**) Ti 2p spectra.

The surface chemical composition and valence state of the samples were determined by X-ray photoelectron spectroscopy (XPS). From Figure 11A, signals corresponding to Ti, O, and Li elements were clearly detected in the samples. The signal of C 1s was also observed, which is typically attributed to adventitious carbon contamination commonly present in XPS analysis and used for binding energy calibration. After tannin modification, no characteristic peaks attributable to tannin (e.g., significantly enhanced carbon signals) were observed in the XPS survey spectra. This is most likely because tannin, as an organic additive, was completely decomposed and removed during the subsequent calcination process at high temperature, leaving no detectable residual signals in the final products.

Figure 11B displays the XPS spectra of the Li 1s orbital. The Li 1s signal of TA-Li_2_TiO_3_ (curve c) is similar to that of the Li_2_TiO_3_ (curve d). Notably, the intensity of the Li 1s peak decreases significantly after acid pickling (curve b: TA-H_2_TiO_3_), indicating that most of the Li^+^ ions are replaced by H^+^ during the acid pickling process. After Li^+^ adsorption (curve a: TA-Li_x_H(_2-x)_TiO_3_), the Li 1s peak only partially recovers, with its intensity remaining much lower than that of the TA-Li_2_TiO_3_. This observation suggests that the Li^+^ ions cannot completely replace the residual H^+^ in the crystal lattice during adsorption, and a portion of H^+^ remains in the structure, preventing full restoration of the initial Li content.

Figure 11C represents the composition of O 1s, from which it can be seen that before acid pickling, the peak at 529.62 eV is ascribed to the Ti-O bonds. While this peak moves 0.46 eV and 0.29 eV respectively to the direction of high binding energy after pickling and adsorption, and the peaks are located at 530.08 eV and 529.91 eV respectively. The change trend of O 1s is consistent with that of Ti^4+^ in Figure 11D. The variation in Ti-O binding energy is caused by the change in crystal structure. The sign at 531.61 eV in the sample before pickling is attributed to surface hydroxyl (-OH) species [47].

Figure 11D shows the Ti 2p spectra. For the TA-Li_2_TiO_3_ sample before acid pickling (curve c), the peaks at 463.80 eV and 458.12 eV correspond to the Ti 2p_1_/_2_ and Ti 2p_3_/_2_ orbitals, respectively. The intensity ratio between the two peaks is approximately 2:1, consistent with the inherent rule of spin–orbit coupling, indicating that Ti predominantly exists in the IV oxidation state in the sample [48]. After acid pickling (curve b) and Li^+^ adsorption (curve a), the Ti 2p_3_/_2_ characteristic peaks shift toward higher binding energies, with shifts of approximately 0.46 eV and 0.39 eV, respectively, relative to the pristine sample. This phenomenon can be attributed to the partial substitution of Li^+^ by H^+^ in the Li and LiTi_2_ layers during acid treatment. Since the ionic radius of H^+^ is much smaller than that of Li^+^, lattice contraction occurs, shortening the Ti–O bond length. This leads to a decrease in the electron cloud density around Ti atoms (as oxygen electrons tend to bond more strongly with H^+^), thereby increasing the binding energy of Ti core-level electrons. After Li^+^ adsorption, some Li^+^ re-enters the lattice and replaces part of the H^+^, partially restoring the crystal structure. However, because H^+^ is not completely displaced, the lattice contraction is only partially relieved; consequently, the Ti 2p_3_/_2_ binding energy remains higher than that of the original unacidified sample. This indicates that the Li^+^/H^+^ exchange process is a reversible ion-exchange process dominated by chemisorption. During adsorption, the basic framework of HTO remains stable, with no Ti^4+^ reduction or lattice collapse observed, demonstrating that the modified ion sieve possesses a basis for chemical stability under cyclic use.

#### 3.2.3. Adsorption Kinetics

LiOH solution with a Li^+^ concentration 2 g/L was employed as the solution to be adsorbed. At 25 °C, TA-H_2_TiO_3_ and LiOH solutions at a solid–liquid ratio of 2 g:100 mL were taken for adsorption for 24 h to reach adsorption equilibrium. The adsorption kinetics of TA-H_2_TiO_3_ (1 g: 1 L) was studied through pseudo first order (Equation (5)) and pseudo second order (Equation (6)) kinetics equations [38], and the corresponding linear fittings were performed.

Pseudo first-order kinetics equation:

(5) $$\mathrm{lg}\left(Q_{e}-Q_{t}\right)=\mathrm{lg}Q_{e}-\frac{k_{1}}{2.303}t$$

Pseudo second-order kinetics equation:

(6) $$\frac{t}{Q_{t}}=\frac{1}{k_{2}Q_{e}^{2}}+\frac{1}{Q_{e}}t$$

where *Q*_e_ is the adsorption capacity (mg·g^−1^) at the adsorption equilibrium, *Q*_t_ is the corresponding adsorption capacity (mg·g^−1^) at time *t*, and *k*_1_ (h^−1^) is the adsorption constant of the pseudo first order kinetic equation, while *k*_2_ (g·mg^−1^·h^−1^) is the adsorption constant of the pseudo second-order kinetic equation.

**Figure 12:**
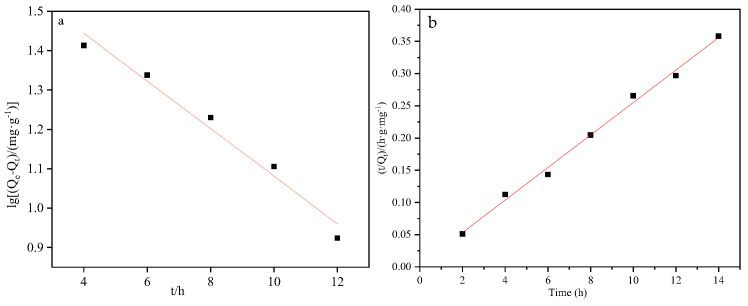
Linear fitting curves of (**a**) pseudo-first-order kinetic and (**b**) pseudo-second-order kinetic.

As shown in Figure 12 and Table 2, the pseudo-second-order kinetic model yields a significantly higher coefficient of determination (R^2^ = 0.99354) than that of the pseudo-first-order model (R^2^ = 0.97411). Moreover, the calculated equilibrium adsorption capacity (Qe = 38.71 mg·g^−1^) is in close agreement with the experimental value (39.13 mg·g^−1^). These results indicate that the pseudo-second-order kinetic model better describes the adsorption kinetics of TA-HTO, suggesting that chemisorption is the dominant adsorption mechanism for this material.

#### 3.2.4. Adsorption Isotherm

**Figure 13:**
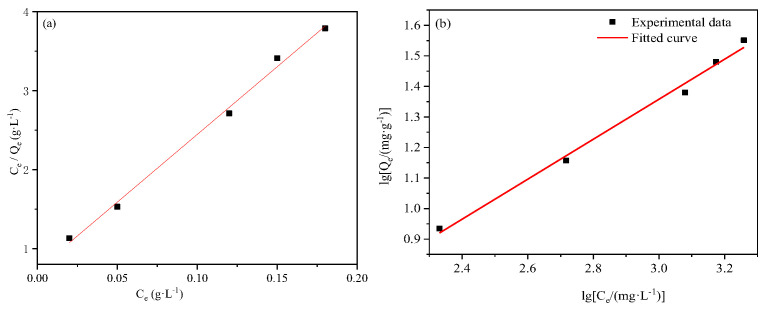
Adsorption isotherms of TA-H_2_TiO_3_ (**a**) Langmuir model; (**b**) Freundlich model.

LiCl solutions with different concentrations were used as the solutions to be adsorbed, and pH of solutions were adjusted with NaOH solution. TA-H_2_TiO_3_ and LiCl solutions were taken for adsorption with the solid–liquid ratio of 0.2 g:60 mL, and the adsorption isotherms of the hydrophilic modified lithium ion sieve were studied. The adsorption models of an adsorbent usually satisfy the Langmuir (Equation (7)) and Freundlich models (Equation (8)), which are given as follows.

(7) $$\frac{C_{e}}{Q_{e}}=\frac{1}{Q_{m}}C_{e}+\frac{1}{K_{L}Q_{m}}$$

(8) $$\mathrm{lg}Q_{e}=\frac{1}{n}\mathrm{lg}C_{e}+\mathrm{lg}K_{F}$$

where *C*_e_ represents the residual Li^+^ concentration in the solution at adsorption equilibrium (mg·L^−1^), *Q*_e_ is the equilibrium adsorption capacity (mg·g^−1^), *Q*_m_ presents the theoretical maximum adsorption capacity (mg·L^−1^), K_L_ is the Langmuir adsorption constant, and n and K_F_ are the Freundlich adsorption constants. The linear fitting results are shown in Figure 13, and the calculation results are given in Table 3. From Figure 13a, it can be observed that when the Langmuir model is used to analyze the results at adsorption equilibrium, *C*_e_ and *C*_e_/*Q*_e_ show a good linear relationship (R^2^ = 0.99378). While lg*C*_e_ and lg*Q*_e_ exhibit an approximate linear relationship when the Freundlich model is used for analysis, and R^2^ is 0.99214. The Langmuir model yields a slightly higher R^2^ value, suggesting a marginally better fit. The adsorption process of Li^+^ by TA-H_2_TiO_3_ can be better described by the Langmuir model compared to the Freundlich model. The theoretical maximum adsorption capacity is calculated to be 58.37 mg·g^−1^, and the specific data is given in Table 3. This indicates that the adsorption of Li^+^ onto TA-H_2_TiO_3_ tends to follow a monolayer coverage mechanism on a homogeneous surface.

## 4. Conclusions

In this work, TA-Li_2_TiO_3_ with a uniform porous structure and enhanced hydrophilicity was successfully prepared by tannin-assisted modification at a solid-to-liquid ratio of 1 g:1 L and calcination at 750 °C. The contact angle decreased from 34.8° to 14.4°. The BET specific surface area increased from 2.6 to 17.7 m^2^/g. The enhanced hydrophilicity and porous structure not only provided more accessible active sites to accelerate interfacial mass transfer, but also alleviated structural stress concentration caused by lattice strain during repeated acid washing and adsorption cycles, owing to the uniformly dispersed particles and abundant mesopores. Meanwhile, the good crystallinity under optimized conditions ensured the structural stability of the ion sieve during subsequent acid washing and adsorption cycles. The elution rate of TA-Li_2_TiO_3_ reached 98.15%, significantly higher than that of unmodified Li_2_TiO_3_. The maximum adsorption capacity reached 39.13 mg/g, an increase of 6.41 mg/g. The adsorption isotherm was better described by the Langmuir model, and the adsorption kinetics followed the pseudo-second-order model, indicating a chemisorption-dominated monolayer adsorption process. In summary, tannin modification systematically enhanced the intrinsic structural stability and adsorption-elution performance of the material by improving crystallinity, increasing specific surface area, enhancing hydrophilicity, and promoting reversible ion exchange.

Although these findings demonstrate the potential of TA-Li_2_TiO_3_ for lithium recovery from aqueous resources, it should be noted that this study was conducted at a laboratory scale using synthetic LiOH solutions. Further pilot-scale studies using real salt-lake brines are needed to evaluate its long-term cycling stability, selectivity in complex ionic matrices, and economic viability. In addition, future work should focus on developing granulated or membrane-immobilized forms of the adsorbent to facilitate continuous-flow operation and large-scale industrial applications.

## Figures and Tables

**Table 1:** The structural information (surface area, pore volume, pore diameter) of samples.

Sample	Surface Area (m^2^/g)	Pore Volume (cm^3^/g)	Pore Diameter (nm)
TA-Li_2_TiO_3_ (1 g:1 L)	17.7	0.0073	5.64
Li_2_TiO_3_	2.6	0.0011	6.46

**Table 2:** Kinetic parameters for Li^+^ adsorption of TA-H_2_TiO_3_.

Sample	Q_e_/(mg·g^−1^)	Pseudo-First-Order		Pseudo-Second-Order	
		k_1_/h	R^2^	k_2_/(g·mg^−1^·h^−1^)	R^2^
TA-H_2_TiO_3_	38.71	0.0975	0.97411	0.0196	0.99354

**Table 3:** Types and parameters of isothermal adsorption of TA-H_2_TiO_3_.

Langmuir			Freundlich		
*Q_m_*/(mg·g^−1^)	*K_L_*/(L·mg^−1^)	R^2^	*n*	*K_F_*/(L·g^−1^)	R^2^
58.37	17.1324	0.99378	1.5273	0.2476	0.99214

## Data Availability

The original contributions presented in this study are included in the article. Further inquiries can be directed to the corresponding authors.
